# Trade-offs between environmental and economic factors in conversion from exotic pine production to natural regeneration on erosion prone land

**DOI:** 10.33494/nzjfs512021x163x

**Published:** 2021

**Authors:** Suzanne M. Lambie, Shaun Awatere, Adam Daigneault, Miko U.F. Kirschbaum, Michael Marden, Tarek Soliman, Raphael I. Spiekermann, Patrick J. Walsh

**Affiliations:** 1 Manaaki Whenua – Landcare Research, Private Bag 3127, Hamilton 3240, New Zealand; 2 School of Forest Resources, University of Maine, 5755 Nutting Hall, 04469, USA.; 3 Manaaki Whenua – Landcare Research, Private Bag 11052, Palmerston North 4442, New Zealand; 4 Research Associate with Manaaki Whenua - Landcare Research, 48 Hillview Terrace, Mangapapa, Gisborne 4010, New Zealand.; 5 Manaaki Whenua – Landcare Research, Private Bag 92170, Auckland 1142, New Zealand; 6 US Environmental Protection Agency, 1200 Pennsylvania Ave, NW, Washington, DC 20460, USA.

**Keywords:** Biophysical tree modelling, cost-benefit modelling, erosion susceptibility, kānuka, mānuka, *Pinus radiata*

## Abstract

**Background:**

Some of New Zealand’s exotic pine (*Pinus radiata* D.Don) forests were planted for erosion mitigation but cultural, legislative, environmental, and profitability limitations in some parts of the landscape have led to reassessment of their suitability. There is limited information to support landowner decisions on the viability of natural regeneration of native forest post-pine-harvest.

**Methods:**

We evaluated scenarios of post-harvest natural regeneration, compared to remaining in pine production, using erosion susceptibility determined from historical occurrence of landslides, gullies and earthflows, biophysical growth modelling of mānuka–kānuka (*Leptospermum scoparium-Kunzea ericoides* (A.Rich) Joy Thomps.) shrubland using the process-based CenW model, and cost-benefit analyses using NZFARM with two land use change scenarios, at two levels of erosion mitigation ± honey profits.

**Results:**

In our study area, the Gisborne Region (North Island of New Zealand), ~27% of the land has moderate–very high susceptibility to landslides, 14–22% a high probability of contributing material to waterways, and 19% moderate–very high gully erosion susceptibility. Pines grow 10 times faster than naturally regenerating mānuka–kānuka shrubland, but mānuka–kānuka is used for honey not wood production. Natural regeneration resulted in losses of $150–250 ha^−1^ yr^−1^ compared to the current profitability of pine production. Honey production offset some reduction in pine revenue, but not fully. Thus, the viability of shifting from pines to native forest is highly dependent on landowner impetus and value for non-market ecosystem services (such as cultural and biodiversity values) provided by native forest.

**Conclusions:**

A mosaic of land uses within a property may sufficiently offset income losses with other benefits, whereby highly erosion-prone land is shifted from rotational pine forest production to permanent native forest cover with honey production where possible. At the regional scale in Gisborne, the conversion of the most highly susceptible land under production forestry (315–556 ha) to natural regeneration has the potential for wider benefits for soil conservation reducing erosion by 1–2.5 t yr^−1^ of sediment facilitating achievement of cleaner water aspirations and habitat provision.

## Introduction

Some owners of exotic pine (*Pinus radiata* D.Don) forests in New Zealand aspire to convert parts of their holdings to native forest using natural regeneration after pine harvest. The viability of converting from profitable pine production to natural regeneration is unclear and the lack of information on costs and opportunities associated with adopting this conversion strategy inhibits informed decision-making by landowners.

Much of New Zealand was cleared of native forest during Polynesian and European colonisation (McGlone 1983; Guild & Dudfield 2010); however, some cleared areas were highly susceptible to erosion (McGlone 1989; Rhodes 2001; Guild & Dudfield 2010). Non-native conifers were planted by the NZ Government in the 1960s as a fast-growing solution to mitigate erosion. Government-owned forests in the Gisborne Region were privatised in the 1980s, shifting these forests from conservation plantings to rotational pine production (Rhodes 2001). NZ’s pine forest industry covers >1.7 million hectares (6.3% of total area; 155,600 ha in the Gisborne Region) and contributes ~$3.55 billion to the New Zealand economy (Forest Owners Association & Ministry for Primary Industries 2019).

The forestry industry has positive and negative societal and environmental impacts. For example, there has been strong employment growth (Nixon et al. 2017) and enhanced value through carbon credits (Akpa et al. 2016; Evison 2008; Jindal et al. 2008) but sediment and debris deposition during storm events damages downstream environments (Grant & Wolff 1991; Landcare Research & Scion 2017; Arnold 2018). There are currently additional environmental and safety regulatory pressures on production forestry (e.g., National Environmental Standard for Production Forestry, National Policy Statement for Freshwater Management, National Policy Statement for Indigenous Biodiversity, Health and Safety at Work Act 2015) that may prove untenable for future wood production in some existing areas of pine forest (Clark 2017; Richards 2017). Some of these areas may be more suitable for retirement and natural regeneration post-pine-harvest. A second pathway to permanent carbon sink and natural regeneration is via unharvested pine stands (Climate Change Commission 2021). This scenario has not been addressed in our work as there is insufficient data on the extent of this practice (Forbes & Norton 2021) in NZ, and the perceived negative impacts of pine seedlings and tree fall (Gibson 2021) require further investigation.

Mānuka–kānuka shrubland, composed of mānuka (*Leptospermum scoparium* J.R.Forest & G.Forst) and kānuka (*Kunzea ericoides* (A.Rich) Joy Thomps.) is often the first stage of natural regeneration in the Gisborne Region (Williams 1983; Newsome 1987; Wilson 1994; Funk et al. 2009; Overdyck & Clarkson 2012) providing permanent erosion mitigation and possible revenue from mānuka honey. Mānuka–kānuka shrubland accounts for ~70% of regenerating forests in New Zealand (Ministry for Primary Industries 2017b) and native forests have greater potential for erosion mitigation than production pines as clear-cut forest management creates a window of vulnerability for sediment and debris transportation during storm events after harvest (Phillips et al. 2012; Lambie et al. 2018). This is supported by Aburto et al. (2021) who showed native *Nothofagus* forest had greater erosion mitigation than *Pinus radiata* production forests in Chile. The scale of work undertaken by Aburto et al. (2021) is comparable with Bergin et al. (1993; 1995) and Marden and Rowan (1993) in New Zealand. While native forest will likely have greater erosion mitigation and cleaner water benefits compared to pine production, retirement of pine estates will decrease income from wood production (Hall et al. 2017) and other avenues of income may need to be sourced such as honey, biodiversity, or carbon.

To facilitate informed decision-making by landowners and regulatory authorities, we assessed the viability of converting erosion-prone land currently planted in pines to natural regeneration using high-resolution erosion susceptibility modelling, biophysical modelling of mānuka–kānuka shrubland, and an ecosystem services cost-benefit model. The cost-benefit model included scenarios with varying levels of erosion susceptibility and with and without honey production to assess economic viability of a range of scenarios.

## Methods

### Study Area

**FIGURE 1:**
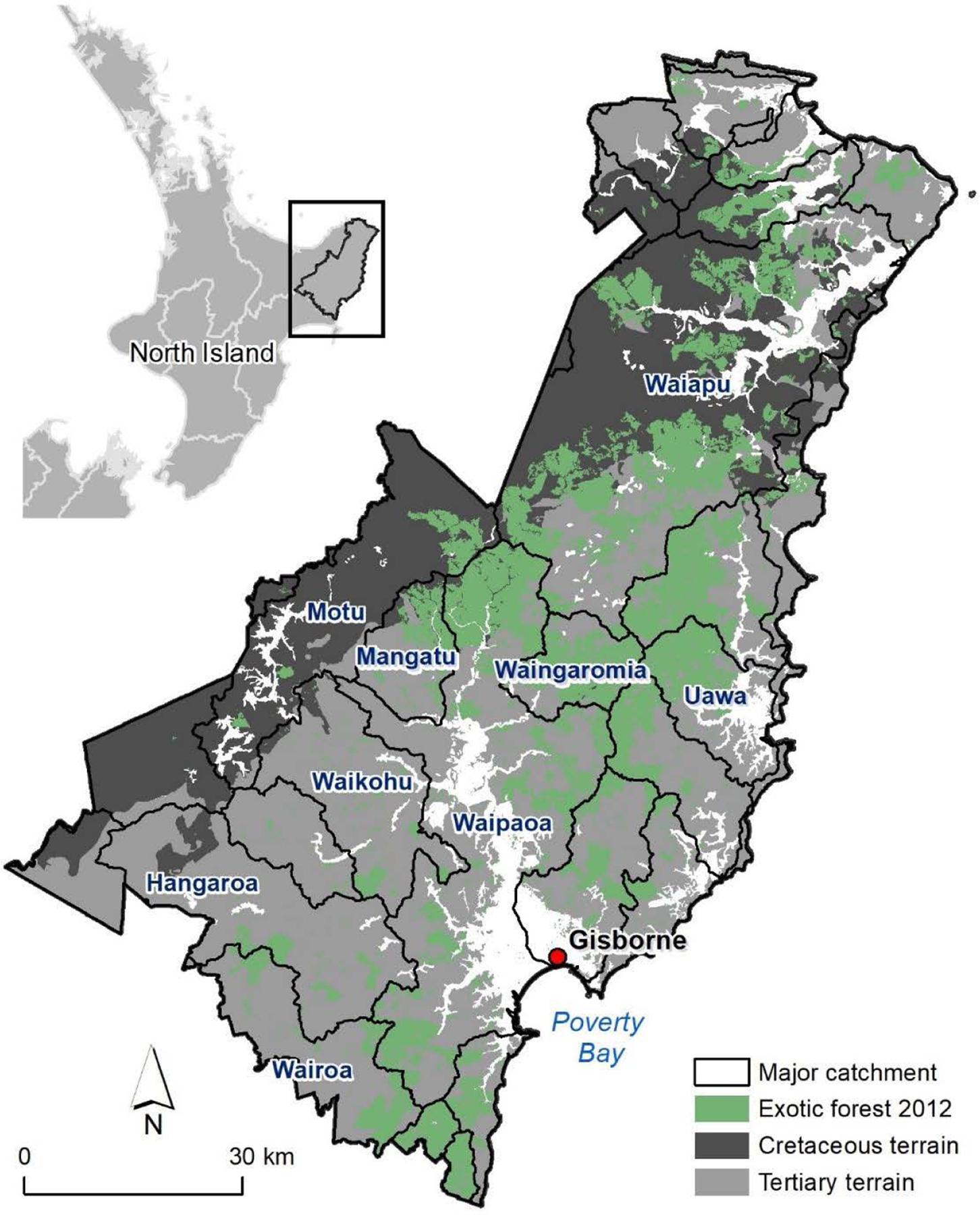
Geological terrain in the Gisborne Region and exotic forest cover based on the Land Cover Database of New Zealand (Landcare Research 2015).

Our study area was the Gisborne Region (835,500 ha) of Te Ika-a-Māui, North Island of Aotearoa, NZ (Fig. 1), which has the greatest proportion of highly erosion-prone land in New Zealand (Ministry for Primary Industries 2017c). The Gisborne Region has two main lithological terrains (Fig. 1): Cretaceous terrain (29% of the region) which is highly indurated and affected by earthflows and rotational slumping, and Tertiary terrain (61% of region), which is less deformed but affected by landslides (Black 1980). Both terrains are susceptible to gully formation (Pearce et al. 1987; Derose et al. 1998; Fuller & Marden 2011).

### Landslide susceptibility and hillslope connectivity

Erosion susceptibility assessments for shallow landslides, gully erosion, and earthflows were undertaken at a 1:25,000. Erosion susceptibility under existing pine plantation forest was assessed. Although different areas of forest were of different ages, we assumed a uniform vegetation cover as the inventory of landslide scars was mapped on pastoral land. Therefore, we were unable to investigate variation in land cover type, and the assessment was limited to lithology and topographic factors. Selection of conditioning factors was based on an understanding of the geomorphic process being assessed. Lithology, slope, and aspect were selected as erosion-conditioning factors, and all have direct physical process relevance for slope stability. The conditioning factors were assessed using bivariate statistics to determine weights and combined in a landslide-index (Juang et al. 1992; Ruff & Czurda 2008; Sciarra et al. 2017) to characterise landslide susceptibility, with rainfall being the cause of landslides. Conditions under which past landslides occurred were used to predict occurrence of future landslides where similar site conditions prevail (Varnes 1984; Soeters & Van Westen 1996; Aleotti & Chowdhury 1999). Spatial distribution of landslides on Tertiary-aged terrain was collated from Betts et al. (2017) who mapped 3,164 landslide scars on similar terrain in the Manawatu region, and Veld and de Graaf (1990) who compiled an inventory of 576 landslide scars on Arai Matawai and Emerald Hills stations ~20 km southwest of Gisborne City following Cyclone Bola (1988). For the Cretaceous terrain, a landslide inventory from the Eastern Ruahine Ranges was used where we assumed that the greywacke bedrock is representative of the Cretaceous terrain found within the Gisborne District. The influence of conditioning factors in susceptibility indices was weighted as per Betts et al. (2017), where slope classes were defined and weights determined by calculating ‘densities’, or the prior probability of failure within each slope class, based on the location of mapped landslides:

(1) $$P_{\text{Prior}}=P\left\{S\right\}=\frac{Npix_{j}(\text{Slide})}{Npix_{j}(\text{Total})}$$

where *P*_*Prior*_ = P{S} is the conditional probability of having a landslide *S and Npix*_*j*_ *(Slide)* are the number of pixels with landslides in slope class, and *Npix*_*j*_ *(Total)* are the total number of pixels in slope class *j*. The prior probabilities are normalised to create weights *W*_*j*_ within the range of 0–1:

(2) $$\left\{W_{j}\right\}=\frac{\left[P(S_{j})-P(S_{min})\right]}{\left[P(S_{max})-P(S_{min})\right]}$$

Scar-slope relationships were established using a national 15-m digital elevation model (DEM) derived from contour data applied separately for Tertiary and Cretaceous terrains. The prior probability of landslide for the Tertiary terrain was calculated as the mean of the scar-slope relationships, and for the Cretaceous terrain, a single scar-slope relationship was used. Slope aspect was calculated using the 15-m DEM to create nine aspect classes, and the relationship with aspect was assessed on a pixel basis. The conditional probability for aspect was calculated in the same way as it was for slope. Finally, the two conditioning factors (slope gradient for each lithology type and aspect) were multiplied to calculate the landslide susceptibility index, which was then classified into five categories - none, low, moderate, high, and very high using equal weights.

### Hillslope connectivity

Connectivity between hillslopes and waterways assessed if a landslide would deliver sediment to a waterway (Dymond et al. 2006). The 15-m DEM was used to estimate the likely flow-path of material generated by a landslide, its flow direction, and potential intervening accumulation zones, to determine whether sediment and/or slash could potentially enter a stream network. If the flow path encountered any significant flat land (consecutive pixels below four degrees of slope), the source pixel was tagged as ‘non-connected’, as we assumed material to be deposited on the flat terrain before reaching the stream. Otherwise, the pixel was tagged as ‘connected’. Connectivity was determined for parcels of land with moderate–very high landslide susceptibility as these land parcels were most likely to be retired from pine forest production and reverted to natural forest that affords longer-term erosion control.

### Gully and earthflow susceptibility

Gully inventories mapped from aerial photography flown in 1957 (1:15,000) and 1997 (1:26,000) were combined (Marden et al. 2012) to identify actively eroding gullies, and the units (with an average size of 110 ha) within the New Zealand Land Resource Inventory (NZLRI) (Landcare Research 2010) that would be most likely to develop gullies. The combined mapped gully layers were then intersected with the NZLRI units. A matrix was used to create gully susceptibility classes (1–5) based on the number of existing gullies (frequency), the potential for future gullies to develop within each NZLRI unit, and the proportion (% of area) of each unit affected by current and past gullying (magnitude). Of the 2,097 NZLRI units in the Gisborne Region identified in the 1970s as having ‘present gully erosion’, 126 units showed no evidence of gullies when mapped in 1957 and 1997 (Marden et al. 2012). On the assumption that gullies were present in these units in the 1970s, the gully severity ranking assigned to these units at the time of the original mapping of LRI units was adopted.

Earthflow susceptibility assessment draws on the NZLRI (Landcare Research 2010), which includes an erosion severity ranking for earthflows as mapped in 1990 and assigned to classifications between ‘none’ to ‘very high’.

### Biophysical modelling

Provision of erosion mitigation by forests is strongly linked to its growth characteristics, which are influenced by climatic, soil and tree species factors. Natural regeneration in New Zealand often begins with mixed mānuka (*Leptospermum scoparium* and kānuka (*Kunzea ericoides* var. *ericoides*) stands (Stephens et al. 2005). Although natural regeneration can begin with broadleaf/podocarp species (e.g., Cameron 1960), there is no published information on the growth and structure of early growth podocarp forest. We, therefore, used mānuka–kānuka shrubland as a representative of natural regeneration as this is the most likely pathway in the Gisborne Region (Funk et al. 2009; Ministry for Primary Industries 2017b).

Growth simulations of mānuka for the Gisborne Region were assessed using a comprehensive dataset of tree growth parameters (e.g., height and diameter at breast height) from across New Zealand from Payton et al. (2010), the New Zealand National Vegetation Survey Databank (NVS) and other unpublished datasets.

Carbon (C) accumulation in mānuka–kānuka shrubland was estimated using model simulations with the physiological model CenW Version 5.0 (Kirschbaum 1999a). CenW has been used extensively to predict the growth of pine stands (e.g., Kirschbaum 1999a, b; Kirschbaum & Watt 2011; Kirschbaum et al. 2012). The model and its source code are available at: http://www.kirschbaum.id.au/Welcome_Page.htm, with a list of relevant equations available at http://www.kirschbaum.id.au/CenW_equations.pdf. For the present work, it was parameterised against the observations from Payton et al. (2010) to simulate the growth of mānuka based on external environmental drivers (temperature and rainfall), stand-internal factors (stand density), and weed competition.

In CenW, weeds are modelled as a separate entity that competes with the main species of interest for nutrients, water, and radiation. Normally, the tree canopy eventually overtops weeds and out shades them, with the time course depending on the initial biomass of weeds and overstorey species and the parameters that define the competitive properties of the weed layer. Here, we assumed a maximal height of the weed layer of 0.5 m and initial weed biomass as 1500 kgDM ha^−1^ and mānuka–kānuka stands as 200 kgDM ha^−1^. These parameters correspond to primary competition by a well-established grass understory.

**FIGURE A1:**
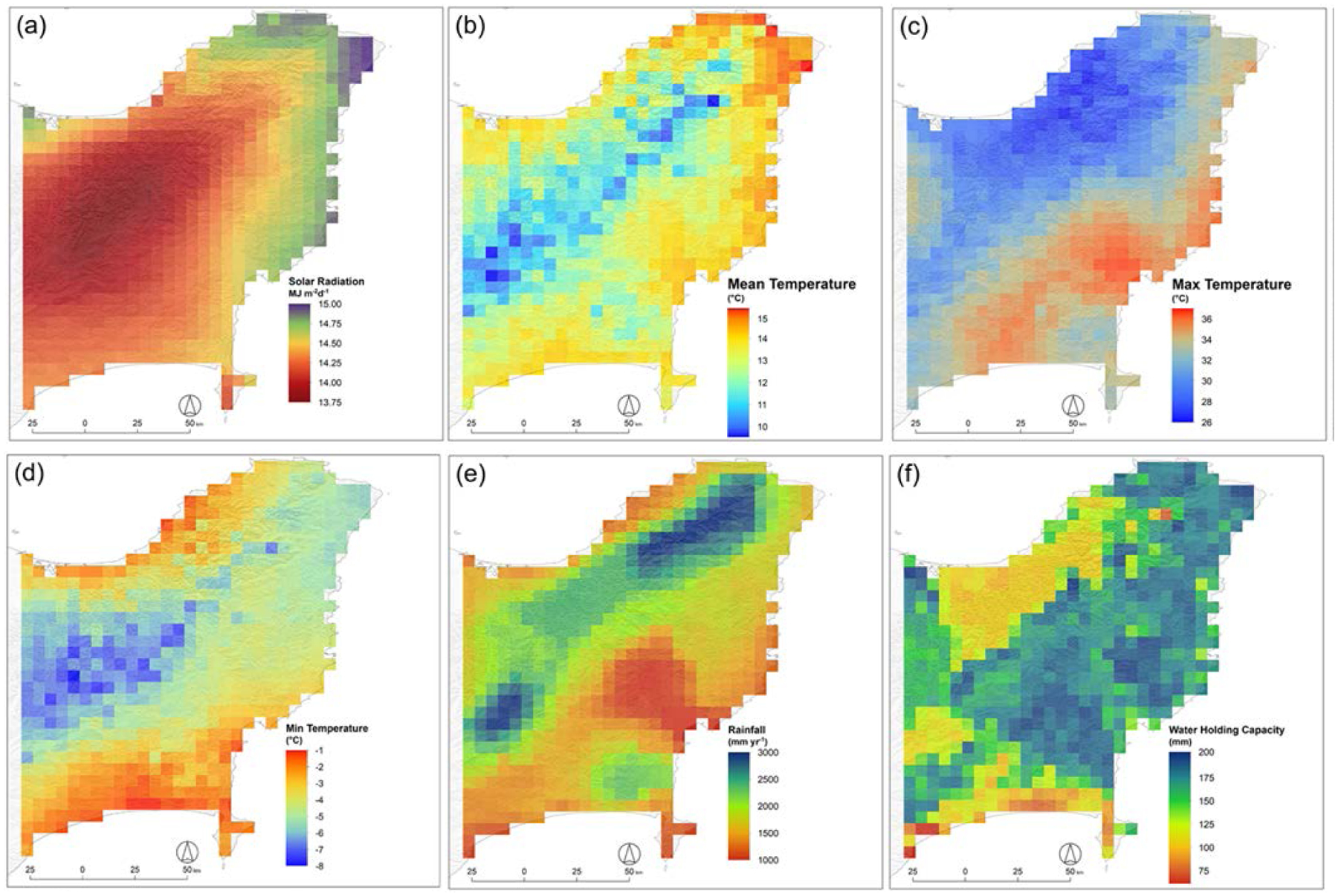
Key environmental variables and soil characteristics of the Gisborne Region, radiation (a); mean temperature (b); recorded maximum (c); and minimum temperatures over a 20-year period (d); rainfall (e); and soil water-holding capacity (f). Climatic data refer to the 1980–1999 period, with panels giving either the averages recorded over that period (a, b, e) or the absolute extremes recorded over that period (c, d). All data are shown at 0.05-degree resolution.

CenW can be run over periods of many decades with an underlying daily simulation time step. It simulates stand properties and dynamics, such as leaf-area development, stand height, basal area development, litter-fall and exchange of both water and carbon dioxide based on daily inputs of minimum and maximum temperature, solar radiation, rainfall, and vapour pressure (Kirschbaum 1999a). It also requires estimates of site fertility, soil water-holding capacity, and silt and sand fractions as a measure of soil texture. We used 20 years of daily weather input data from the Virtual Climate Station Network (VCSN; National Institute of Water and Atmospheric Research Ltd, Appendix 1). Daily VCSN data were estimated on a 0.05° latitude/longitude grid (Tait et al. 2006; Tait 2008; Tait & Liley 2009) as described by Kirschbaum and Watt (2011). Soil water-holding capacity and the percentage of silt plus clay were obtained from the National Soils Database (Landcare Research 2020). Characteristic climate variables for the Gisborne Region are presented in Appendix Figure A1.

Predictions of different measures of growth were fitted to independent observations from 69 stands located at 52 distinct locations throughout New Zealand (Payton et al. 2010), with growth measures mostly consisting of total stand biomass at respective ages inferred from ring counts. We also used the observed distribution of mānuka–kānuka throughout New Zealand to derive functions to constrain the environmental performance of mānuka–kānuka under more extreme climatic conditions than the sites sampled by Payton et al. (2010).

Some parameter estimates were based on the earlier modelling work of Whitehead et al. (2004) and Whitehead and Walcroft (2005) and the observations of Burrows et al.^1^ and Burrows^2^. Other specific information on stand allometric properties was sourced from Scott et al. (2000) and leaf nitrogen concentrations from Ross et al. (2009). The range of parameter values was further constrained to remain within physiologically plausible bounds to retain the physiological integrity of the simulations. The modelling procedure was similar to that described by Kirschbaum and Watt (2011) for pine growth.

### Cost-benefit analysis

A cost-benefit analysis using a combination of a spatially explicit agri-environmental economic land-use model (NZFARM) (Daigneault et al. 2018) and other non-market valuation methods was used to monetise changes in land use based on categories of erosion susceptibility. Landslide susceptibility categories 4 (moderate susceptibility with high waterway connectivity), 6 (high susceptibility with high waterway connectivity), 8 (very high susceptibility with high waterway connectivity), and land highly prone to gullying (as per Spiekermann and Marden 2018) were the focus of model scenarios. The model scenarios had two levels of landsliding susceptibility with more categories included in Scenario 2 and consistent gully susceptibility across all scenarios. The erosion susceptibility scenarios were tested with and without honey production as a potential offset to losses in wood profits. Scenario 1, landslide classes 6+8 (high and very high landslide susceptibility with high waterway connectivity) and high gully category converted from pines to native forest with honey production. Scenario 2, landslide classes 4+6+8 (moderate, high, and very high landslide susceptibility and high waterway connectivity) and high gully category converted from pines to native forest with honey production. Scenario 3, same as Scenario 1, without honey production and Scenario 4, same as Scenario 2, without honey production.

Model scenarios were compared to the ‘Baseline’ scenario, where all identified land remains in pine plantation forestry. The baseline was established using a land use map of the Gisborne Region (AgriBase; Asure Quality 2020) and the New Zealand Land Cover Database (Landcare Research 2015). The model includes assessments of production impacts including profit from land converted from pine forestry, profit from land converted to mānuka honey or related production, value of the land, and planting/native afforestation costs and environmental impacts including carbon cycling and water quality.

The analysis was conducted over 62 years to reflect two cycles of 30-year pine rotation (Hockey & Page unpublished^3^; Ministry for Forestry 1994). Since the benefits and costs occur over different time periods, the net present value was determined at two discount rates (4 and 6%; Te Tai Ōhanga 2018) to calculate overall impacts:

(3) $$\text{NPV}=\sum_{t=1}^{62}\frac{{\left(\text{Impact}\right)}_{t}}{{\left(1+r\right)}^{t}}$$

where *t* is each year (up to 62), and *r* is the discount rate.

Treasury New Zealand recommended the use 4 and 6% rates to convert future values to present values as money in the future is worth less than the same amount in the present (Te Tai Ōhanga 2018). The range of discount rates also reflects the different objectives of landowners. For instance, the lower end of the range might reflect the objective of achieving intergenerational equity as some landowners (e.g., Māori) might not be driven solely by profits but by other societal goals such as intergenerational resource sustainability as well as maintaining cultural and spiritual benefits for future generations (Goulder & Williams 2012; Harmsworth and Awatere 2013).

Net present value of economic returns for the pine forestry sector were calculated as follows:

(4) $$NPV_{t}=\frac{\left(p\times Vol_{t}-\text{Cos}t_{t}\right)}{{\left(1+r\right)}^{t}}$$

where *p* is the log price (Te Uru Rākau 2020), *Vol* is the log volume harvested in each year (from CenW; Kirschbaum & Watt 2011), and *Cost* is the fixed and variable costs of production. Fixed and variable costs including logging, cartage, roading, and growing costs were derived from the literature (Olssen et al. 2012). All future values of the production function were then discounted to get total present values. We finally derived the equal annual equivalent (EAE) from the estimated net present value to compare the annual economic returns from pine forestry with other land uses. Economic returns from mānuka honey produced from naturally regenerating mānuka–kānuka shrublands were based on Daigneault et al. (2015).

Environmental factors (*E*_*i*_) including nutrient leaching, erosion, and carbon sequestration were monetised following estimation on a per hectare basis (*γ*^*env*^), as impacted by soil type, land cover, and land use. By aggregating the per hectare values of these parameters across the land area under pine (*X*), we could estimate the total environmental factor outputs from pine forestry:

(5) $$\sum \gamma^{\text{env}}X=E_{i}$$

Surficial and stream bank erosion were simulated using the NZEEM model (Dymond et al. 2010), and nutrient values obtained (e.g., Parfitt et al. 1997; Lilburne et al. 2010). GHG emissions were sourced from MPI carbon look-up tables (Ministry for Primary Industries 2017a). To reflect the impact of land conversion from pine production to native forest on the environmental outputs, Equation 5 was modified to:

(6) $$\sum \gamma^{\text{env}}(X-Z)+\mu^{\text{env}}Z=E_{i}$$

where *Z* is the area of the land converted to native forest. The parameter *γ*^*env*^ specifies the environmental impacts of pine forest after accounting for land conversion, while *μ*^*env*^ describes the impact of native forest on the environmental factors.

Several non-market techniques have been developed to place a price on changes in water quality, including hedonic pricing (Boyle et al. 1999), recreation demand (Massey et al. 2006), and stated preferences (Moore et al. 2018). Of those techniques, estimates from stated preference studies capture the widest range of people and values, both use and non-use, in their application depending on the scope of the study. We therefore focus on stated preference values and used willingness to pay (WTP) for improved water quality. To monetise water quality improvements associated with each scenario, a benefit transfer for people’s WTP for reductions in nutrient leaching was used, which is common technique for determining the value of an ecosystem service (e.g., Aguilar et al. 2018; Huber and Finger 2020; Tian et al. 2020). Nutrient leaching values were sourced from Takatsuka et al. (2009), where a choice experiment was used to estimate monetary values of improvements in water quality as well as other ecosystem services. Based on the values estimated by Takatsuka et al. (2009), a linear transfer function was estimated (although a non-linear function produced comparable results). Estimates are corrected for differences in income and nominal dollars between the original and current study. To value carbon sequestration, lookup tables were first used to calculate changes in carbon for each scenario (Ministry for Primary Industries 2017b) using a natural logarithm regression model for 50–62 years (r^2^ = 99.99). Carbon sequestration values were then assumed to be $25 tCO2e^−1^, based on prices from recent transactions of the New Zealand Emission Trading Scheme (ETS). The change in erosion was monetised using a mid-range estimate of $3 t^−1^ sediment based on two independent estimates of $1 t^−1^ (Dymond et al. 2012) and $6.50 t^−1^, where the upper value includes only avoided flood damage and water treatment costs (Barry et al. 2014).

Annual honey profits in mānuka–kānuka shrublands vary largely depending on honey quality ranging between $98 ha^−1^ for low unique mānuka factor (UMF) to $1000 ha^−1^ for high UMF (Walsh et al. 2017). These profit estimates were based on a production per hive of 30 kg and 35 kg and a price per kg of $26.30 and $40 for the $98 ha^−1^ and $1000 ha^−1^, respectively (Burke 2015; Wetere 2015). The variation in profits was due to differences in mānuka productivity, and capital and operating costs. UMF is a rating system designed to ensure purity, quality, and authenticity of mānuka honey, and the higher the UMF rating the higher the monetary value of the honey. While no spatially explicit data is available on where high UMF honey has been produced, recent research predicted that soil quality, rainfall, climate, and genotype are critical variables in determining the UMF level in a particular area. To identify areas suitable for high UMF honey, we therefore used temperature and precipitation prediction equations (Watt et al. 2012) to define the probability of occurrence of mānuka–kānuka shrubland as per Walsh et al. (2017). The probabilities were summed and assigned to NZFARM polygons based on an area-weighted average to create an index describing the relative likelihood of occurrence of mānuka–kānuka. Based on conversations with local farmers and associations, the top 10% of polygons was assumed to be areas suitable for high UMF honey and therefore allocated a profit of $1000 per hectare, while the rest of the polygons were assumed to produce low UMF and were allocated a profit of $98 per hectare.

Much of the value of agricultural land is tied to the profits associated with it, so we assume that the opportunity cost of removing land from production is reflected in the change in the NPV of future profits. NZFARM outputs change in net revenue represent the central opportunity costs of the change from pine production to native forest with and without honey production. We were able to monetise several notable impacts associated with changes in land use. However, it is important to note that there are a number of other impacts that we were not able to quantify or monetise (Table 1). Biodiversity was quantified as per Walsh et al. (2019) and presented as ‘restored significance’ as per Mason et al. (2012) and Carswell et al. (2015), but were not monetised and therefore not included in the economic analysis. Further, the impacts of weed control and competition were not able to be included in the analysis due to limited support information.

## Results

### Erosion susceptibility

Incidences of landslides increased markedly on slopes >16° in Tertiary terrain and often delivered sediment and forest slash into the nearest watercourse. In contrast, on the Cretaceous terrain, shallow landslides are predominantly restricted to the steep flanks of the Raukumara Range. Slope aspect was an important factor for landslide susceptibility in our study area, as found by others (e.g., Yalcin & Bulut 2007; Galli et al. 2008; Ruff & Czurda 2008; van Westen et al. 2008). We found a disproportionate number of landslides on north to north-east facing slopes, and landslide density on north facing slopes was 50% higher than average.

**FIGURE 2:**
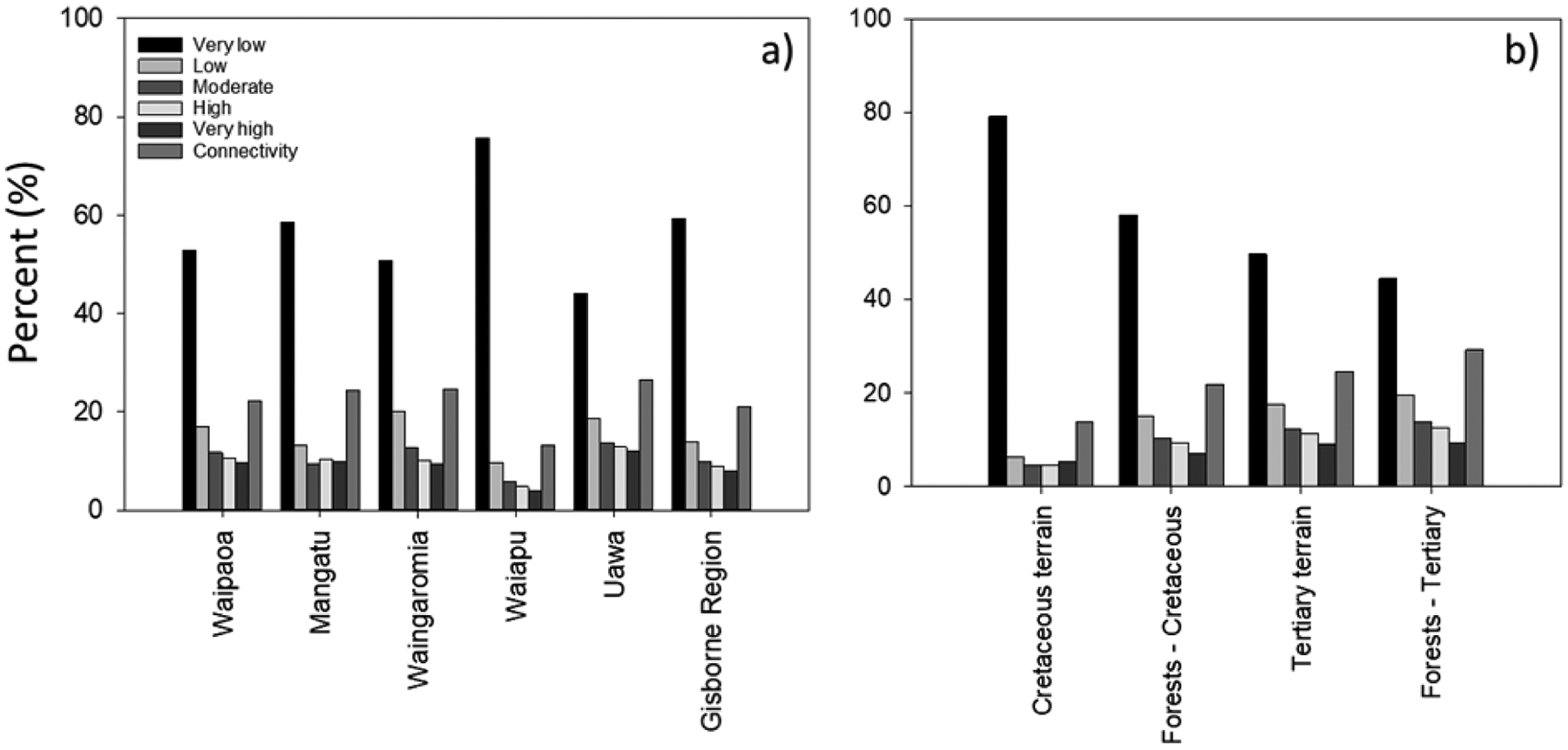
Landslide susceptibility, as a percentage of: (a) major catchments and Gisborne Region; and (b) for the area of Tertiary and Cretaceous terrains, including pine forest areas only. For landslide susceptibility, the percentage of hill country with moderate to very high susceptibility and potential to deliver sediment (connectivity) to waterways is also shown.

In the Gisborne Region, 73.2% (545,700 ha) had very low–low landslide susceptibility and 21.1% (157,600 ha) had moderate–very high susceptibility. Moderate–very high areas had landslides that were likely to deliver sediment and other material into nearby waterways. Of the two terrain types, Tertiary underlies the largest proportion of the Gisborne Region and landslide susceptibility was very low–low on 67.4% of hill country areas, and moderate–very high on the remaining 32.6% of hill country, of which approximately 165,000 ha (22%) of hill country slopes are directly connected to waterways (Fig. 2). Overall, 125,000 ha (24.7%) of hill country slopes are moderate–very highly susceptible and have potential for landslides and/or anthropogenic disturbances resulting in sediment, and any associated woody debris, entering a water course.

In comparison, Cretaceous terrain has slopes that were generally less steep and landslide susceptibility was very low–low on 85.5% of hill country areas, and moderate–very high on 14.5% (34,800 ha) of remaining hill country areas, of which 13.7% (32,700 ha) of hill country slopes were directly connected to waterways (Fig. 2).

Land under pine forests has higher levels of landslide susceptibility than the average for the whole region (Fig. 2) as they were established on erosion prone land to mitigate erosion. A larger proportion of the current forest estate is on Cretaceous than Tertiary terrain (Landcare Research 2015). However, land occupied by pine forests on Tertiary terrain has a much higher proportion of land susceptible to landslides (35.9% in classes moderate–very high) than the pine forests on Cretaceous terrain (26.8%).

**FIGURE 3:**
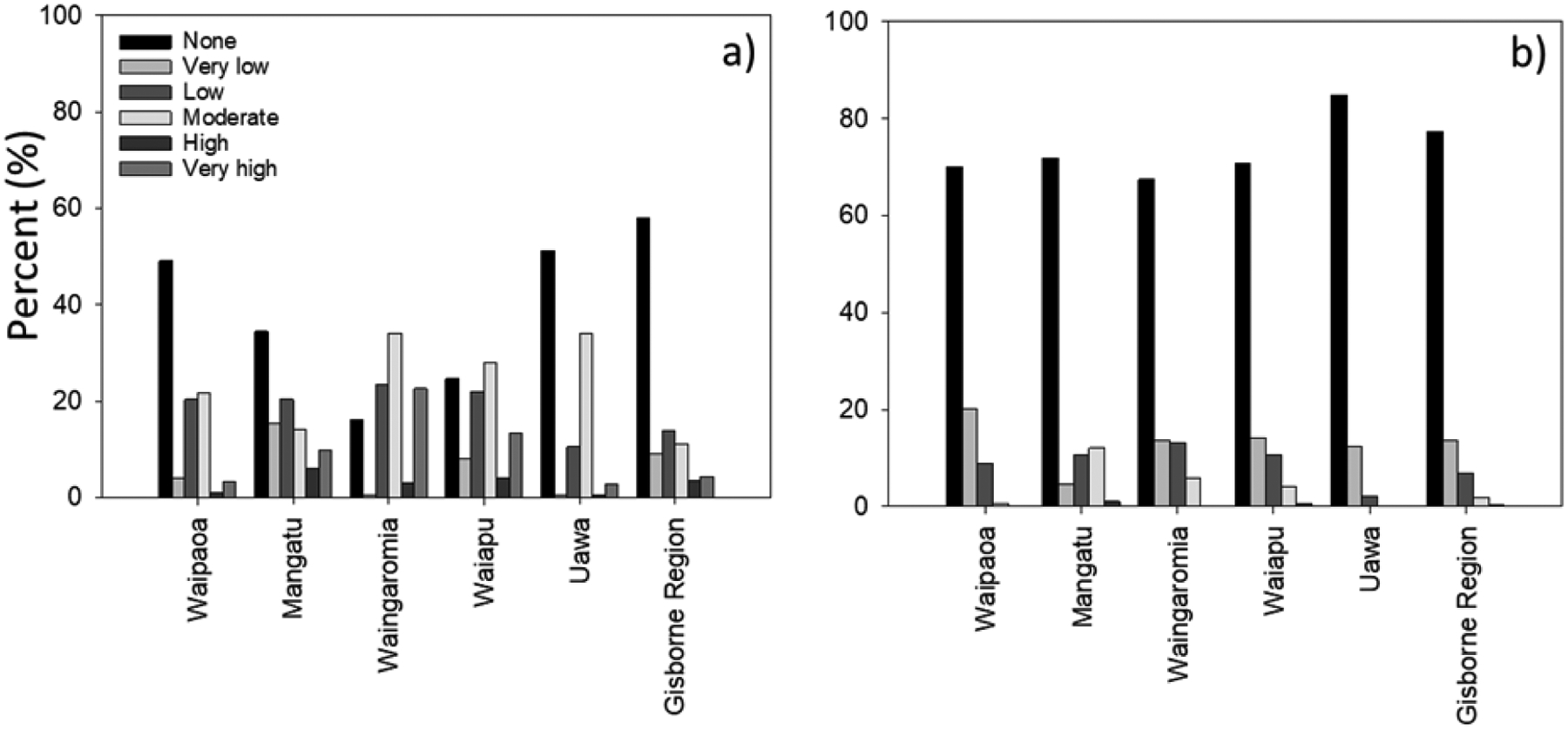
Erosion susceptibility for: (a) gully; and (b) earthflow erosion, as a percentage of major catchments and Gisborne Region.

Within the Gisborne Region, 19% of the region is moderate–very high for gully erosion susceptibility (Fig. 3a). 22.5% of hill country is classified as susceptible to earthflow erosion, with just 8.8% classed as moderate–very high susceptibility (Fig. 3b) and is associated with areas dominated by mudstone or crushed argillite.

### Tree growth

**FIGURE 4:**
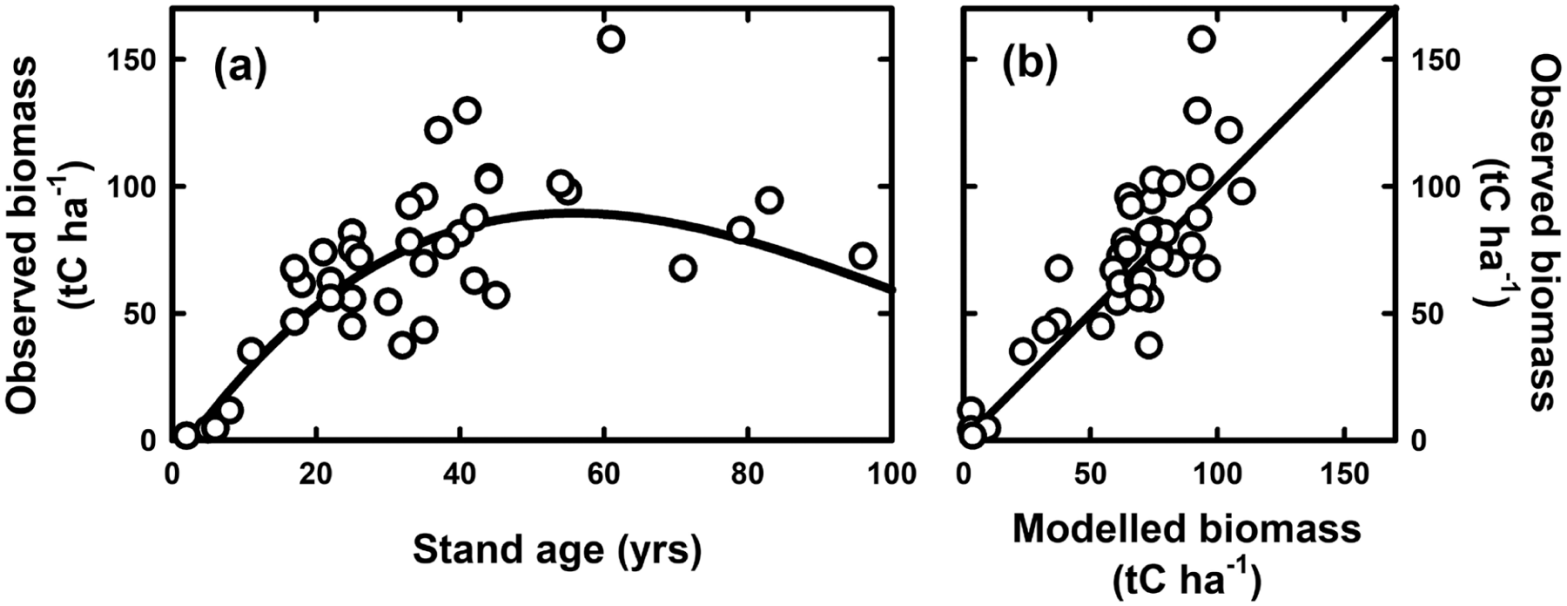
Observed and modelled growth potential of mānuka-kānuka stands of different ages, showing observed (symbols) and modelled (solid curve) biomass (a) and observed biomass plotted against modelled data (b). Observed data are from Payton et al. (2010) and simulations from CenW.

To better understand the growth potential of mānuka–kānuka stands, we parameterised the CenW model against the observed data of Payton et al. (2010) (Fig. 4). Stands showed some moderate growth potential over the first 20 years, reaching stand biomass of about 50 tC ha^−1^. Growth rates then slow, with peak stand biomass typically reached by about 50 years. Stands then tend to degenerate over further time or are invaded by taller trees that then dominate. The observed growth patterns could be captured well by the CenW simulations (Fig. 4b), with a calculated model efficiency of 0.65.

Growth potential of stands is strongly affected by initial stand density, which interacts with weed competition. Once seedlings over-top the competing grass layer, a period of rapid growth can commence. Stand growth rate tends to be reduced through self-thinning that reduces the number of living trees with associated loss of stand biomass. Growth rates are therefore typically only a fraction of those achieved by well-managed *P. radiata* stands (cf. Kirschbaum et al. 2011).

**FIGURE A2:**
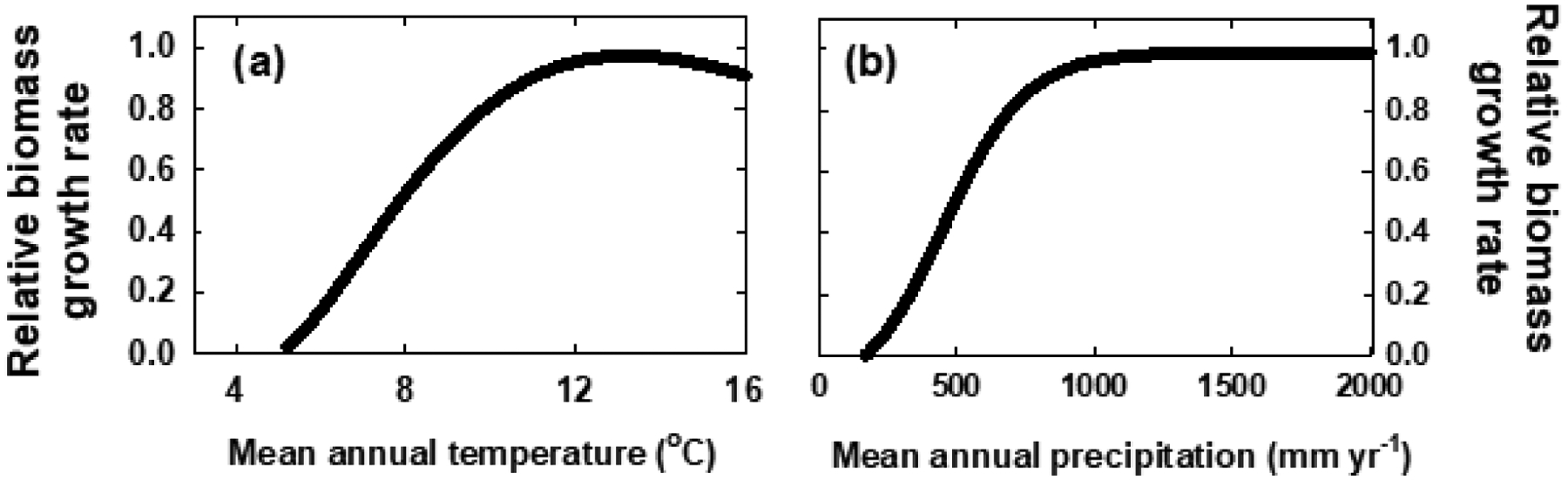
The relative growth response of mānuka–kānuka stands for different mean annual temperatures (a); and mean annual rainfall (b). These relationships have been developed based on analysis of the data of Payton et al. (2010) and the observed distribution of mānuka–kānuka stands across New Zealand.

Analysis of growth rates (Payton et al. 2010) and the patterns of temperature and rainfall in relation to the natural distribution of mānuka–kānuka stands across the country were used to estimate tree growth in response to environmental drivers. We had insufficient information to make a distinction between the growth of mānuka and kānuka and assumed the same environmental limitations for mānuka stands and mānuka–kānuka shrublands. Mānuka–kānuka stands require mean annual temperatures of more than ~5°C (Appendix Fig. A2a), and growth increases rapidly with increasing temperature to about ~12°C (Fig. A2a). Similarly, mānuka–kānuka stands require annual rainfall > ~300 mm yr^−1^ (Appendix Fig. A2b), and growth increases steeply for optimal performance at ~800–1000 mm yr^−1^, with no further growth response at higher rainfall. However, it is not currently known whether extremely high rainfall beyond 2,000 mm yr^−1^ adversely affects the growth and persistence of stands.

**FIGURE 5:**
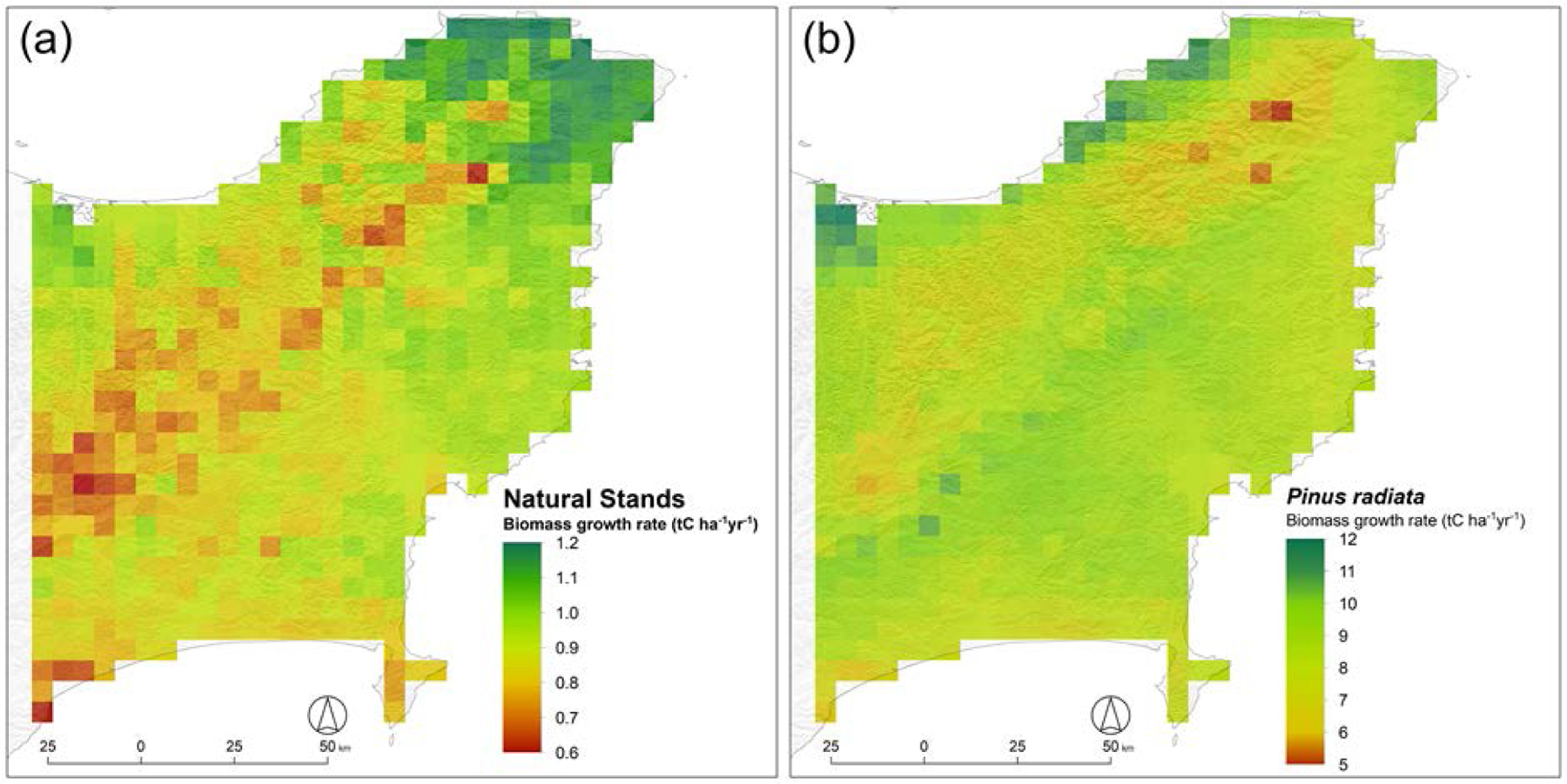
Simulated growth over 20 years of: (a) total biomass for naturally regenerating mānuka–kānuka stands (100,000 stems ha^–1^); and over 30 years of: (b) total biomass for pine (*P. radiata)* in the Gisborne Region. Note the 10-fold difference in scales between mānuka–kānuka stands and pine.

For the Gisborne Region, simulated growth of mānuka–kānuka was highest in the north-east, where it could reach up to 1.2 tC ha^−1^ yr^−1^, and marginally lower in the coastal regions on both the north and southeast (Fig. 5a). Growth was slightly lower along the higher-elevation inland ridge where cooler temperatures limited growth to 0.7–0.8 tC ha^−1^ yr^−1^. Low values were also obtained for a few southern coastal sites where low precipitation combined with soils with low soil water-holding capacity to cause water-stress limitations.

Pines are capable of much faster biomass and wood accumulation than naturally regenerating shrubland. Stem diameters in naturally regenerating stands are much smaller than for pines because of the much higher stand density of naturally regenerating stands, so that their biomass must be spread over a larger number of stems (Fig. 5). Pine growth was modelled to be highest in the regions with moderately high rainfall, with biomass growth of up to 12 tC ha^−1^ yr^−1^ (Fig. 5b). This corresponds to wood production of 350–450 tDM ha^−1^ over a 30-year rotation (data not shown). In contrast, growth in the high-rainfall Raukumara Range ridge running from north-east to south-west was slightly lower with biomass growth of only 7–8 tC ha^−1^ yr^−1^. Unlike mānuka (Stephens et al. 2005), pines generally do not grow well in regions with excessive rainfall (Kirschbaum & Watt 2011).

### Cost-benefit analysis

The estimated area of pine forests for conversion in each scenario was 315 and 556 ha, or 0.33 and 0.59% of the total planted pine area in the study area. In the first two scenarios, which included honey production in native forest areas, 1–2 ha was classified as high-UMF production (Table 2) and were estimated to have high economic returns, while the remainder of the land was assumed to produce lower-value honey with poorer economic returns. Seven and 15 ha were estimated to incur additional expenses for successful native regeneration (Table 2), including purchasing plants and planting costs. The remaining land within the scenarios was predicted to have sufficient landscape and geographic factors to permit natural regeneration (Walsh et al. 2019) and was likely to be mānuka–kānuka shrubland initially (Bray et al. 1999; Funk et al. 2009; Ministry for Primary Industries 2017b). There was lower reduced carbon storage and erosion under all four scenarios compared to the baseline (Table 2). There were also some changes in biodiversity and water quality but given the size of those changes and the lack of usable nonmarket values, they were difficult to monetise. Restored significance, as an estimate of biodiversity improvements, was 324±20 ppb for all scenarios.

Overall, the monetised NPV of converting from pine in each of our scenarios is negative (Table 3). Converting pine forestry land reduces profits from production and the land value, and the two combined can represent significant decreases. Although revenue can be obtained from other enterprises based on natural afforestation, such as mānuka honey or oil, these are not as profitable as growing pines. However, there were some environmental benefits that could not be monetised and therefore were omitted from the NPV calculations. Highly erosion-prone land is usually on steep slopes and the analysis of profits and land values may be overestimated (Table 3).

Across the four scenarios, losses in NPV ranged between $3 and $8 million over the 62-year period or ~$150-$250 ha^−1^ yr^−1^ and lost profits from forestry represented the largest costs of conversion. Decreases in carbon sequestration were also notable, as native forest stores less carbon across 62 years than pines. Other social benefits, such as environmental and cultural factors, are more difficult to quantify. Yao et al. (2014) found that there is a willingness of the New Zealand public to pay for enhancement of forest ecosystems for biodiversity provision, but further work is needed to adequately provide values for biodiversity and cultural parameters, which may further offset wood derived profits under our scenarios. Several other categories could be neither quantified nor monetised. In terms or overall impacts, these omitted categories would likely decrease the overall negative impacts of our scenarios. These omitted values may provide compelling reasons to promote native afforestation. Cultural impacts, for instance may justify the loss in forestry profit is some cases.

It is also difficult to calculate potential changes in employment resulting from our modelled land use conversions. However, since the scenarios we analysed affect only small land areas, the employment impact should be minor. Larger conversions would likely result in proportionately larger employment changes with wider regional economic impacts.

## Discussion

Both plantation pine and natural regeneration have long-term benefits for catchments and communities. However, clear-felling of pine stands can lead to introduction of sediment and woody debris to waterways during storm events (Marden et al. 2006; Imaizumi et al. 2008; Payn et al. 2015; Issaka & Ashraf 2017; Spiekermann & Marden 2018). Erosion susceptibility is inherently variable within catchments and geological terrains suggesting that land cover should ideally be as diverse as the landforms to create a mosaic of land uses across a property or catchment.

### Economic impacts of conversion from pine production to native forest

If assessing the viability of converting pines to natural regeneration on a purely economic basis, pines are considerably more profitable than natural regeneration, even when other market benefits of natural regeneration are included. The costs of conversion would be borne by pine forest owners, in terms of lost profit and changes in land value. Conversely, many of the benefits from forests accrue to the general population, such as improvements in local air and downstream water quality, carbon sequestration, and cultural impacts (e.g., Nowak et al. 2012).

Mānuka–kānuka shrubland is often the first phase of natural regeneration (Overdyck & Clarkson 2012), and under suitable circumstances, honey profits may be used to offset some of the losses from retiring land under pines. Scenario 2 may represent the ideal situation whereby the most highly vulnerable parts of the landscape (susceptible to both landslides and gullying) are retired from production forestry and allowed instead to develop a permanent tree cover, with the associated benefit of honey production.

Many historical pine plantations were established in very difficult, steep terrain, often far from urban centres, ports, and mills increasing operational and infrastructural costs (Raymond 2012). These impacts on profits were not included in our analysis and strategies are being developed to improve harvesting efficiency on steep sites, it is likely that profits for these slopes may be underestimated (Raymond 2012, 2014; Amishev et al. 2013). On particularly difficult slopes, trees may remain unharvested, especially during periods of low log prices (Review Panel 2014) and therefore act as a nursery crop of natural regeneration and any profits for these sites will be unrealised.

Weeds can reduce the growth and survival of target species and is an important factor for long-term erosion mitigation and monetary profits. Weeds can initially compete strongly with mānuka–kānuka seedlings for light, water, and nutrients, but once mānuka trees are taller than competing weeds, they gain largely unrestricted access to solar radiation and will out-shade low-stature weeds once the canopy is sufficiently large and dense. Our growth simulations were based on assuming a well-established grass layer as the principal weed competitor. Because of its high initial biomass, grass acts as an effective competitor in the early establishment phase, but because of its low maximum height, mānuka–kānuka seedlings could soon out-shade the grass layer and become the dominant plant type. If shrubs, such as gorse or broom, were the principal competitive weed species, their competitive inhibition of the growth of mānuka–kānuka seedlings could have persisted for longer or could even have prevented the establishment of the shrubland altogether.

These impacts of weeds have been well characterised for pines (e.g., Richardson et al. 1996; Watt et al. 2007), but we are not aware of systematic studies of weed effects on the establishment of mānuka stands. Pine seedlings are another substantial weed problem occurring post-harvest and during the natural regeneration stage. Pine seedlings can have a substantial impact on native species establishment and survival (Marlborough District Council et al. 2016). Further, the costs of pine seedling control can be substantial ($150–500 ha^−1^) and affect the species composition of naturally regenerating forests as different native species differ in their sensitivity to wilding control with herbicide applications (Marlborough District Council et al. 2016; Lambie & Marden 2020).

### Drivers for conversion of pine production to native forest

Land-management decisions are also subject to social influences as well as the economic factors (Miller et al. 2007; Bhandari et al. 2015). Enhancing biodiversity values is a driver for shifts from pine to native forests (Marlborough District Council et al. 2016; Lambie & Marden 2020). Pine forests have good biodiversity values, providing habitat for many plants and animals including threatened species (Brockerhoff et al. 2001; Bremer et al. 2010; Pawson et al. 2010; Michelsen et al. 2014; Berndt & Brockerhoff 2019). Native forests typically contain a greater species richness compared with pine forests, as native forest has a greater range of food sources that are unimpacted by harvest and forest structural diversity providing a greater range of habitat; however, native forest is highly temporally and spatially variable (Clout & Gaze 1984; Díaz et al. 2005; Pawson et al. 2010). As a result, some landowners who highly value these attributes may be willing to reduce their profits from pine revenues.

Biodiversity offsetting is also a potential pathway for recognising the value associated with native forests (Department of Conservation 2014). However, a monetary or currency value associated with biodiversity offsetting in New Zealand remains highly complex and a valuation framework is required (Department of Conservation 2014). Biodiversity offsetting is generally supported through resource management undertaken by Councils, under the umbrella of the Resource Management Act. Substantial work has been undertaken to establish guidance on biodiversity offsetting (Department of Conservation 2014; Maseyk et al. 2018) and it is possible that this mechanism will be further supported under the Resource Management Act reforms and in the future (Ministry for the Environment 2021).

There are also pathways where financial value can be assigned to projects that enhance biodiversity. For example, Green bonds, Green funds, sustainability linked loans and biodiversity credits (Chartres 2021). Biodiversity credits are the most likely pathway by which monetary value associated with increased biodiversity and systems for biodiversity credits are being put in place internationally (e.g., Porras and Steel 2020; NSW Department of Planning, Industry and Environment 2021). Biodiversity credits can be linked to biodiversity offsetting (NSW Department of Planning, Industry and Environment 2021) but require significant development of a framework in which to undertake credit trading and recognition in New Zealand.

Shifts from pine to native forest cover can also be driven by cultural values (Hēnare 2014). Former state-owned forest assets are returning to Māori under Treaty of Waitangi claims and could result in 41% of post-harvest pine forests being Māori-owned (Miller et al. 2007). Māori connect with indigenous forests on a spiritual level associated with whakapapa (genealogy) and kaitiakitanga (guardianship), which provide additional non-economic factors for decisions on land use (Miller et al. 2007) that may be attractive to Māori landowners. In the Gisborne Region much of the most erosion-prone land is on Māori-owned land (Miller et al. 2007), which may provide an added incentive for increasing indigenous forests.

Carbon credits may also drive natural regeneration. However, changing from a fast-growing tree crop such as pine trees to potentially slower growing native trees will reduce carbon stocks and carbon sequestration and ultimately result in less carbon credits compared to pine trees (Kimberley et al. 2014) and is more profitable when converting from pasture to forests. Further, carbon accrual in regenerating forests is calculated generically from look-up tables which are particularly lacking with respect to native species, and do not contain regional or species-specific information (Ministry for Primary Industries 2017b). Carver and Kerr (2017) suggest that updating the tables to include more native tree specific information will facilitate inclusion of native forests in the ETS and therefore greater recognition of these forests for carbon income.

## Conclusions

We suggest a mosaic of land use within a property (or catchment) may be the best overall option, where moderate to highly susceptible land is left to naturally regenerate post-pine-harvest, while pine production is maintained on land less susceptible to erosion. For this multi-land use approach to be considered viable and adopted successfully, landowners will need to understand the erosion susceptibility of their land to create an impetus to shift from pine trees to native forest. This would benefit from support from local and central government agencies with the overall aim to meet cleaner water aspirations. Many of the current mechanisms to encourage erosion mitigation are not applicable to those wanting to shift from pines to native forests and only support planting in previously unforested areas (e.g., One Billion Trees Programme, Erosion Control Funding Programme). Further, the relatively lower amount of carbon credits that native forests accrue relative to pine plantations may also discourage shifting to native species. Overall, these results illustrate the economic value of the pine forestry industry, and the consequent challenges with transitioning to other land uses despite the greater environmental benefits associated with reducing erosion.

## Figures and Tables

**TABLE 1:** Summary of Main Impacts of Land Conversion Scenarios

Main impacts	Monetised	Quantified
*Production-related impacts*		
Profit from land converted from exotic forestry	X	
Profit from land converted to honey or related production	X	
Value of land	X	
Planting/native afforestation costs	X	
*Cultural impacts*		
Cultural Medicines		
Cultural Harvests		
Aesthetic/landscape changes		
*Environmental Impacts*		
Biodiversity		X
Carbon	X	
Water quality	X	
Water quantity		
Recreation		
Threatened or endangered species		
*Health and Community Impacts*		
Injuries		
Changes in unemployment		
Changes in population		
Welfare impacts of native forest proximity		

**TABLE 2:** Model inputs for cost-benefit valuation for four scenarios, two scenarios with high (6) to very high (8) landslide susceptibility and two with moderate (4), high (6), and very high (8) landslide susceptibility, all scenarios included high gully prone land. Scenarios 1 and 3 are the same except for the inclusion of honey in Scenario 1. Scenarios 2 and 4 are the same except for the inclusion of honey in Scenario 2. LS is the landslide susceptibility category, and Δ indicates the change from the baseline scenario.

Component	Scenario 1	Scenario 2	Scenario 3	Scenario 4
	LS 6+8, +gully + honey	LS 4+6+8, +gully + honey	LS 6+8, +gully	LS 4+6+8, +gully
Total area converted (ha)	315	556	315	556
High UMF area (ha)	1.04	2.18	0.00	0.00
Active afforestation area (ha)	7.56	14.46	7.56	14.46
Stored carbon Δ (tC)	−13,347	−23,600	−13,347	−23,600
Erosion Δ (t yr^−1^)	−1,077	−1,968	−1,351	−2,469

**TABLE 3:** Baseline NPV (NZ $) across 62 years and change in NPV for four scenarios (4% and 6% discount rates), two scenarios with high (6) to very high (8) landslide susceptibility and two with moderate (4), high (6), and very high (8) landslide susceptibility, all scenarios included high gully prone land. Scenarios 1 and 3 are the same except for the inclusion of honey in Scenario 1. Scenarios 2 and 4 are the same except for the inclusion of honey in Scenario 2. Where LS is the landslide susceptibility category, ‘seq’ represents sequestration, ‘afforest’ represents afforestation, and Δ indicates the change from the baseline scenario.

Component	Change in NPV from Baseline (NZ$)			
	Scenario 1	Scenario 2	Scenario 3	Scenario 4
	LS 6+8, +gully + honey	LS 4+6+8, +gully + honey	LS 6+8, +gully	LS 4+6+8, +gully
Carbon seq (4%)	−1,427,491	−2,524,004	−1,427,491	−2,524,004
Carbon seq (6%)	−1,373,127	−2,427,881	−1,373,127	−2,427,881
Reduced erosion (4%)	76,638	140,035	96,113	175,621
Reduced erosion (6%)	54,504	99,591	68,354	124,899
Native afforest (4%)	−54,088	−103,375	−54,088	−103,375
Native afforest (6%)	−54,088	−103,375	−54,088	−103,375
Net revenue (4%)	−2,106,090	−3,745,326	−2,886,646	−5,133,173
Net revenue (6%)	−1,497,820	−2,663,621	−2,052,940	−3,650,638
Water quality (4%)	9,871	17,454	9,871	17,454
Water quality (6%)	6,721	11,884	6,721	11,884
Total NPV Δ (4%)	−3,501,160	−6,215,216	−4,262,241	−7,567,477
Total NPV Δ (6%)	−2,863,810	−5,083,402	−3,405,080	−6,045,111

## Footnotes

1 Burrows, L.E. (2010). *Estimate of biomass and leaf area index (LAI) for kānuka at Avoca Station*. [Unpublished report], Lincoln, New Zealand: Landcare Research.

2 Burrows, L., Carswell, F., Brignall-Theyer, M., McKenzie, S. (2009). *Changes in biomass carbon during kānuka shrubland succession to forest*. [Landcare Research Internal Report LC0809/084], Lincoln, New Zealand: Landcare Research.

3 Hockey, M., Page, M. (1983). *Site index data for the Gisborne-East Coast Region. Collated in 1981 and partially revised in 1983*. Wellington, New Zealand: Unpublished Internal New Zealand Forest Service Report.

## Appendix

### Gisborne climate

Solar radiation across the Gisborne region is fairly uniform, with just slightly higher values at the coast and slightly lower values inland at higher elevations (Fig. A1a). Temperature shows greater variation, with mean annual temperatures above 15°C at the north-eastern coastal tip and dropping to below 10°C in the higher inland locations, especially towards the south-west (Fig. A1b, c, d). There is rainfall of 2,500 to over 3,000 mm yr^−1^ in the upland regions, but less than 1,500 mm yr^−1^ in most of the coastal regions, and rainfall of only around 1,000 mm yr^−1^ inland from Gisborne city (Fig. S1e). In terms of temperature extremes, the area near Gisborne city has experienced the greatest temperature extremes of recorded maxima above 35°C, while the northern side of the peninsula has reached only 26–28°C. Absolute minima have generally been lowest in the highland region with values below −7°C, whereas the coastal regions generally remained milder with minima mostly not falling below −3°C, except for a large part of the east coast where temperatures have fallen to below −5°C.

## List of abbreviations

UMF: unique mānuka factor
NPV: net present value

## References

[R1] Aburto F, Cartes E, Mardones O, Rubilar R (2021). Hillslope soil erosion and mobility in pine plantations and native deciduous forest in the coastal range of south-Central Chile. Land Degradation and Development, 32, 453–466. 10.1002/ldr.3700

[R2] Aguilar FX, Obeng EA, Cai X (2018). Water quality improvments elicit consistent willingness-to-pay for the enhancement of forested watershed ecosystem services. Ecosystem Services, 30(Part A), 158–171. 10.1016/j.ecoser.2018.02.012

[R3] Akpa SIC, Odeh IOA, Bishop TFA, Hartemink AE, Amapu IY (2016). Total soil organic carbon and carbon sequestration potential in Nigeria. Geoderma, 271, 202–215. 10.1016/j.geoderma.2016.02.021

[R4] Aleotti P, Chowdhury R (1999). Landslide hazard assessment: summary review and new perspectives. Bulletin of Engineering Geology and the Environment, 58, 21–44. 10.1007/s100640050066

[R5] Amishev D, Basher L, Phillips C, Hill S, Marden M, Bloomberg M, Moore J (2013). New forest management approaches to steep hills. [MPI Technical Paper No. 2014/39], 109 p. Wellington, New Zealand: Minstry for Primary Industries.

[R6] Arnold N (2018). When the rain came for Tolaga Bay. New Zealand Geographer, 152, 10–15.

[R7] Asure Quality. (2020). AgriBase2020. https://www.asurequality.com/services/agribase/ (accessed 31 October 2020).

[R8] Barry LE, Yao RT, Harrison DR, Paragahawewa UH, Pannell DJ (2014). Enhancing ecosystem services through afforestation: How policy can help. Land Use Policy, 39, 135–145. 10.1016/j.landusepol.2014.03.012

[R9] Berndt LA, Brockerhoff EG (2019). Effects of land cover type on carabid beetles (Coleoptera: Carabidae) of the Canterbury foothills, New Zealand. New Zealand Journal of Forestry Science, 49. 10.33494/nzjfs492019x54x

[R10] Bergin DO, Kimberley MO, Marden M (1993). How soon does regenerating scrub control erosion? New Zealand Journal of Forestry, 38, 38–40.

[R11] Bergin DO, Kimberley MO, Marden M (1995). Protective value of regenerating tea tree stands on erosion-prone hill country, East Coast, North Island, New Zealand. New Zealand Journal of Forestry Science, 25, 3–19.

[R12] Betts H, Basher L, Dymond J, Herzig A, Marden M, Phillips C (2017). Development of a landslide component for a sediment budget model. Environmental Modelling and Software, 92, 28–39. 10.1016/j.envsoft.2017.02.003

[R13] Bhandari KP, Aryal J, Darnsawasdi R (2015). A geospatial approach to assessing soil erosion in a watershed by integrating socio-economic determinants and the RUSLE model. Natural Hazards, 75, 321–342. 10.1007/s11069-014-1321-2

[R14] Black RD (1980). Upper Cretaceous and Tertiary geology of Mangatu State Forest, Raukumara Peninsula, New Zealand. New Zealand Journal of Geology and Geophysics, 23, 293–312. 10.1080/00288306.1980.10424141

[R15] Boyle KJ, Poor PJ, Taylor LO (1999). Estimating the demand for protecting freshwater lakes from eutrophication. American Journal of Agricultural Economics, 81, 1118–1122. 10.2307/1244094

[R16] Bray JR, Burke WD, Struick GJ (1999). Propagule dispersion and forest regeneration in *Leptospermum scoparium* (mānuka) and *L. ericoides* (kānuka) forests following fire in Golden Bay, New Zealand. New Zealand Natural Sciences, 24, 35–52.

[R17] Bremer LL, Farley KA (2010). Does plantation forestry restore biodiversity or create green deserts? A synthesis of the effects of land-use transitions on plant species richness. Biodiversity and Conservation, 19(14), 3893–3915. 10.1007/s10531-010-9936-4

[R18] Brockerhoff EG, Ecroyd CE, Langer ER (2001). Biodiversity in New Zealand plantation forests: Policy, incentives, and the state of our knowledge. New Zealand Journal of Forestry, 31–37.

[R19] Burke J (2015). High performance manuka plantations. Presented at Rotorua Land Use Opportunities Symposium, 16–17 June 2015.

[R20] Cameron RJ (1960). Natural regeneration of podocarps in the forests of the Whirinaki River Valley. New Zealand Journal of Forestry, 8, 337–354.

[R21] Carswell FE, Mason NWH, Overton JM, Price R, Burrows LE, Allen RB (2015). Restricting new forests to conservation lands severely constrains carbon and biodiversity gains in New Zealand. Biological Conservation, 18, 206–218. 10.1016/j.biocon.2014.11.002

[R22] Carver T, Kerr S (2017). Facilitating carbon offsets from native forests. Wellington, NZ: Motu Economic and Public Policy Research. 10.29310/wp.2017.01

[R23] Chartres A (2021). Valuing biodiversity. Accessed 30 August 2021. https://pureadvantage.org/financing-biodiversity/

[R24] Clark P (2017). Clarky’s *comment* - *February*. Accessed 11 March 2021. https://nz.pfolsen.com/market-info-news/wood-matters/2017/february/clarkys-comment-february/.

[R25] Clout MN, Gaze PD (1984). Effects of plantation forestry on birds in New Zealand. Journal of Applied Ecology, 21, 795–815. 10.2307/2405048

[R26] Daigneault A, Greenhalgh S, Samarasinghe O (2018). Economic impacts of multiple agro-environmental policies on New Zealand land use. Environmental and Resources Economics, 69, 763–785. 10.1007/s10640-016-0103-6

[R27] Daigneault A, Wright W, Samarasinghe O (2015). Economic analysis of land use opportunities in Maniapoto rohe. [Landcare Research Contract Report LC2415], 60 p. Te Kuiti, New Zealand: Maniapoto Māori Trust Board.

[R28] Department of Conservation (2014). Guidance on biodiversity offsetting in New Zealand. Wellington, NZ: Department of Conservation.

[R29] Derose RC, Gomez B, Marden M, Trustrum NA (1998). Gully erosion in Mangatu Forest, New Zealand, estimated from digital elevation models. Earth Surface Processes and Landforms, 23, 1045–1053. 10.1002/(SICI)1096-9837(1998110)23:11<1045::AID-ESP920>3.0.CO;2-T

[R30] Díaz IA, Armesto JJ, Reid S, Sieving KE, Willson MF (2005). Linking forest structure and composition: avian diversity in successional forests of Chiloé Island, Chile. Biological Conservation, 123(1), 91–101. 10.1016/j.biocon.2004.10.011

[R31] Dymond JR, Ausseil A-GE, Ekanayake JC, Kirschbaum MUF (2012). Tradeoffs between soil, water, and carbon - A national scale analysis from New Zealand. Journal of Environmental Management, 95, 124–131. 10.1016/j.jenvman.2011.09.019 22115517

[R32] Dymond JR, Ausseil AG, Shepherd JD, Buettner L (2006). Validation of a region-wide model of landslide susceptibility in the Manawatu-Wanganui region of New Zealand. Geomorphology, 74, 70–79. 10.1016/j.geomorph.2005.08.005

[R33] Dymond JR, Betts HD, Schierlitz CS (2010). An erosion model for evaluating regional land-use scenarios. Environmental Modelling and Software, 25, 289–298. 10.1016/j.envsoft.2009.09.011

[R34] Evison D (2008). The impact of carbon credits on New Zealand radiata pine forestry profitability. New Zealand Journal of Forestry, 53, 42.

[R35] Forbes A, Norton D (2021) Transitioning exotic plantations to native forest: A report on the state of knowledge. [MPI Technical Report No: 2021/22], 37 p. Wellington, New Zealand: Ministry for Primary Industries.

[R36] Forest Owners Association, Ministry for Primary Industries. (2019). Fact and figures 2018/2019: New Zealand Plantation Forest Industry. 63p. Wellington, New Zealand: Ministry for Primary Industries.

[R37] Fuller IC, Marden M (2011). Slope-channel coupling in steepland terrain: A field-based conceptual model from the Tarndale gully and fan, Waipaoa catchment, New Zealand. Geomorphology, 128, 105–115. 10.1016/j.geomorph.2010.12.018

[R38] Funk J, Field C, Kerr S, Trotter C (2009). Modeling the impact of Carbon Farming on a New Zealand landscape. PhD Thesis, Chapter II. Stanford University: Palo Alto, CA.

[R39] Galli M, Ardizzone F, Cardinali M, Guzzetti F, Reichenbach P (2008). Comparing landslide inventory maps. Geomorphology, 94, 268–289. 10.1016/j.geomorph.2006.09.023

[R40] Gibson E (2021). *73 million trees ‘ not nearly* enojugh’, *says company using pine to nurture native forest*. Stuff, accessed 26 August 2021. https://www.stuff.co.nz/environment/climate-news/124826669/73-million-trees-not-nearly-enough-says-company-using-pine-to-nurture-native-forest

[R41] Goulder LH, & Williams III RC (2012). The choice of discount rate for climate change policy evaluation. Climate Change Economics, 3, 1250024. 10.1142/S2010007812500248

[R42] Grant GE; Wolff AL (1991). Long-term patterns of sediment transport after timber harvest, western Cascade Mountains, Oregon, USA. In: Peters NE, Walling DE (Eds.), Sediment and stream water quality in a changing environment: trends and explanation, [Proceedings of the Vienna IAHS symposium, Vienna, Austria. IAHS Publication No. 203], (pp 31–40). Oxfordshire, United Kingdom: International Association of Hydrological Sciences.

[R43] Guild D, Dudfield M (2010). A history of fire in the forest and rural landscape in New Zealand - Part 2, post 1830 influences, and implications for future fire management. New Zealand Journal of Forestry, 54, 31–38.

[R44] Hall D, Lindsay S, Judd S (2017). Permanent forest bonds: a pioneering environmental impact bond for Aotearoa New Zealand. [Working paper 17/01], 45 p. Wellington, New Zealand: Institute for Governance and Policy Studies.

[R45] Harmsworth GR, & Awatere S (2013). Indigenous Māori knowledge and perspectives of ecosystems. In: Dymond J (Ed.), Ecosystem services in New Zealand-conditions and trends (*pp* 274–286). Lincoln, New Zealand: Manaaki Whenua Press.

[R46] Hēnare M (2014). Iwi signal end to pine plantations. http://www.uabsknowledge.ac.nz/en/research-and-comment/research-and-analysis/iwi-signal-end-to-pine-plantations.html (Accessed 3 February 2020).

[R47] Huber R, Finger R (2020). A meta-analysis of the willingness to pay for cultural services from Grasslands in Europe. Journal of Agricultural Economics, 71, 357–383. 10.1111/1477-9552.12361

[R48] Imaizumi F, Sidle RC, Kamei R (2008). Effects of forest harvesting on the occurence of landslides and debris flows in steep terrain of central Japan. Earth Surfaces Proceses and Landforms, 33, 827–840. 10.1002/esp.1574

[R49] Climate Change Comission (2021). Ināia tonu nei: a low emissions future for Aotearoa 2021. Wellington, NZ: Climate Change Comission.

[R50] Issaka S, Ashraf MA (2017). Impact of soil erosion and degradation on water quality: a review. Geololgy, Ecology, and Landscapes, 1, 1–11. 10.1080/24749508.2017.1301053

[R51] Kimberley M, Bergin D, Beets P (2014). Carbon sequestration by planted native trees and shrubs. Hamilton, NZ: Tane’s Tree Trust.

[R52] Jindal R, Swallow B, Kerr J (2008). Forestry-based carbon sequestration projects in Africa: Potential benefits and challenges. Natural Resources Forum, 32(2), 116–130. 10.1111/j.1477-8947.2008.00176.x

[R53] Juang CH, Lee DH, Sheu C (1992). Mapping slope failure potential using fuzzy sets. Journal of Geotechical Engineering,118, 475–494. 10.1061/(ASCE)0733-9410(1992)118:3(475)

[R54] Kirschbaum MUF (1999a). CenW, a forest growth model with linked carbon, energy, nutrient and water cycles. Ecological Modelling, 118, 17–59. 10.1016/S0304-3800(99)00020-4

[R55] Kirschbaum MUF (1999b). Modelling forest growth and carbon storage with increasing CO_2_ and temperature. Tellus, 51B, 871–888. 10.1034/j.1600-0889.1999.t01-4-00002.x

[R56] Kirschbaum MUF, Watt MS (2011). Use of a process-based model to describe spatial variation in *Pinus radiata* productivity in New Zealand. Forest Ecology and Management, 262, 1008–1019. 10.1016/j.foreco.2011.05.036

[R57] Kirschbaum MUF, Watt MS, Tait A, Ausseil A-GE (2012). Future wood productivity of *Pinus radiata* in New Zealand under expected climatic changes. Global Change Biology, 18, 1342–1356. 10.1111/j.1365-2486.2011.02625.x

[R58] Lambie SM, Marden M (2020). Transitioning from exotic to native forest through natural regeneration: Benefits and risks. [Landcare Research Contract Report LC3676], 19 p. Napier, New Zealand: Hawkes Bay Regional Council.

[R59] Lambie SM, Marden M, Kirschbaum MUF, Soliman T, Walsh P (2018). Best options for land use following radiata harvest in the Gisborne District under climate change: Literature review. [MPI Technical Paper No: 2018/46]. 76 p, Wellington, New Zealand: Ministry for Primary Industries.

[R60] Landcare Research. (2015). LCDBv4.1 - Land cover database version 4.1, Mainland New Zealand. https://lris.scinfo.org.nz/layer/48423-lcdb-v41-land-cover-database-version-41-mainland-newzealand/ (Accessed 24 January 2020).

[R61] Landcare Research. (2020). National Soils Database. https://soils.landcareresearch.co.nz/soil-data/national-soils-data-repository-and-the-national-soils-database/the-nsdr-improvement-programme/ (Accessed 23 Jaunary 2020).

[R62] Landcare Research (2010). NZLRI North Island, Edition 2 (all attributes). https://lris.scinfo.org.nz/layer/48134-nzlri-north-island-edition-2-all-attributes/ (Accessed 24 January 2020).

[R63] Landcare Research, Scion (2017). Debris flows. https://www.nzfoa.org.nz/resources/file-libraries-resources/environment/factsheets/582-debris/file (Accessed 6 December 2017).

[R64] Lilburne L, Webb T, Ford R, Bidwell V (2010). Estimating nitrate-nitrogen leaching rates under rural land uses in Canterbury. [Environment Canterbury Technical Report R10/127], 37p. Kaikoura, New Zealand: Canterbury Regional Council.

[R65] Marden M, Arnold G, Seymour A, Hambling R (2012). History and distribution of steeplands gullies in response to land use change, East Coast Region, North Island, New Zealand. Geomorphology, 153–154, 81–90. 10.1016/j.geomorph.2012.02.011

[R66] Marden M, Rowan D (1993). Protective value of vegetation on tertiary terrain before and during Cyclone Bola, east coast, North Island, New Zealand. New Zealand Journal of Forestry Science, 23, 255–63.

[R67] Marden M, Rowan D, Phillips C (2006). Sediment sources and delivery following plantation harvesting in a weathered volcanic terrain, Coromandel Peninsula, North Island, New Zealand. Soil Research, 44, 219–232. 10.1071/SR05092

[R68] Marlborough District Council, Department of Conservation, Marlborough Sounds Restoration Trust. (2016). Guidelines for converting pine plantations to native vegetation in the Marlborough Sounds. 11 p. Marlborough, New Zealand: Marlborough District Council.

[R69] Maseyk F, Ussher G, Kessels G, Christensen M, Brown M (2018). Biodiversity offsetting under the Resource Management Act: A guidance document. New Zealand: Biodiversity Working Group.

[R70] Massey DM, Newbold SC, Gentner B (2006). Valuing water quality changes using a bioeconomic model of a coastal recreational fishery. Journal of Environmental Economics and Management, 52, 482–500. 10.1016/j.jeem.2006.02.001

[R71] Mason NWH, Ausseil A-GE, Dymond JR, Overton JM, Price R, Carswell FE (2012). Will use of non biodiversity objectives to select areas for ecological restoration always compromise biodiversity gains? Biological Conservation, 155, 157–168. 10.1016/j.biocon.2012.05.019

[R72] McGlone MS (1983). Polynesian deforestation of New Zealand: A preliminary synthesis. Archaeology in Oceania, 18, 11–25. 10.1002/arco.1983.18.1.11

[R73] McGlone MS (1989). The polynesian settlement of New Zealand in relation to environmental and biotic changes. New Zealournal Journal of Ecology, 12, 115–129.

[R74] Michelsen O, McDevitt JE, Coelho CRV (2014) A comparison of three methods to assess land use impacts on biodiversity in a case study of forestry plantations in New Zealand. The International Journal of Life Cycle Assessment, 19, 1214–1225. 10.1007/s11367-014-0742-1

[R75] Miller R, Dickinson Y, Reid A (2007). Māori connections to forestry in New Zealand. In: Feary S (Ed.) Forestry for indigenous peoples: Learning from experiences in forest industries. (pp. 13–22). Canberra, Australia: Australian National University.

[R76] Ministry for Forestry. (1994). A guide to the East Coast Forestry Project. Wellington, New Zealand: Ministry for Forestry.

[R77] Ministry for Primary Industries. (2017a). Carbon look-up tables for forestry in the emissions trading scheme. Wellington, New Zealand: Ministry for Primary Industries.

[R78] Ministry for Primary Industries. (2017b). A guide to the carbon look-up tables for forestry in the emission trading scheme. 44 p. Wellington, New Zealand: Ministery for Primary Industries.

[R79] Ministry for Primary Industries (2017c). Erosion Control Funding Programme. http://www.mpi.govt.nz/funding-and-programmes/forestry/erosion-control-funding-programme (Accessed 23 January 2020).

[R80] Ministry for the Environment 2021. Overview of the resource management reforms. https://environment.govt.nz/what-government-is-doing/key-initiatives/resource-management-system-reform/overview/#objectives-of-rm-reform (Accessed 26 August 2021).

[R81] Moore C, Guigne D, Dockin C, Maguire KB, Simo NB (2018). Valuing ecological improvements in the Chesapeake Bay and the importance of ancillary benefits. Journal of Benefit-Cost Analysis, 9, 1–26. 10.1017/bca.2017.9 31080702 PMC6510401

[R82] Newsome PFJ (1987). The vegetation cover of New Zealand. Wellington, NZ: Water and Soil Directorate, Ministry of Works and Development.

[R83] Nixon C, Amperle D, Pambudi D, Clough P (2017). Plantation forestry statistics: Contribution of forestry to New Zealand. 83 p. Wellington, New Zealand: New Zealand Institute for Economic Research.

[R84] Nowak DJ, Hirabayashi S, Bodine A, Greenfield E (2014). Tree and forest effects on air quality and human health in the United States. Environmental Pollution, 193, 119–129. 10.1016/j.envpol.2014.05.028 25016465

[R85] NSW Department of Planning, Industry and Environment. (2021). What are biodiversity credits? Accessed 30 August 2021. https://www.environment.nsw.gov.au/topics/animals-and-plants/biodiversity-offsets-scheme/about-the-biodiversity-offsets-scheme/what-are-biodiversity-credits

[R86] Olssen A, Zhang W, Evison D, Kerr S (2012). A forest-profit expectations dataset for New Zealand, 1990–2008. [Motu working paper 12–07], 25p. Wellington, New Zealand: Motu Economic and Public Policy Research. 10.29310/wp.2012.07

[R87] Overdyck E, Clarkson BD (2012). Seed rain and soil seed banks limit native regeneration within urban forest restoration plantings in Hamilton City, New Zealand. New Zealand Journal of Ecology, 36, 177–190.

[R88] Parfitt RL, Percival HJ, Dahlgren RA, Hill LF (1997). Soil and solution chemistry under pasture and radiata pine in New Zealand. Plant and Soil, 191, 279–290. 10.1023/A:1004266000509

[R89] Pawson SM, Ecroyd CE, Seaton R, Shaw WB, Brockerhoff EG (2010). New Zealand’s exotic plantation forest as habitat for threatened indigenous species. New Zealand Journal of Ecology, 3, 342–355.

[R90] Payn T, Phillips C, Basher L, Baillie B, Garrett L, Harrison D, Heaphy M, Marden M (2015). Improving management of post-harvest risks in steepland plantations. New Zealand Journal of Forestry, 60, 3–6.

[R91] Payton IJ, Barringer J, Lambie S, Lynn I, Forrester G, Pinkney T (2010). Carbon sequestration rates for post-1989-compliant indigenous forests. [Landcare Research Contract Report LC0809/107], 35 p. Lincoln, New Zealand: Landcare Research.

[R92] Pearce AJ, O’Loughlin CL, Jackson RJ, Zhang XB (1987). Reforestation: On site effects on hydrology and erosion, eastern Raukumara Range, New Zealand. Forest Hydrology and Watershed Management, 167.

[R93] Phillips C, Marden M, Basher L (2012). Plantation forest harvesting and landscape response - what we know and what we need. New Zealand Journal of Forestry, 56, 4–12.

[R94] Porras I, Steele P (2020). Making the market work for nature: How biodiversity credits can protect biodiversity and reduce poverty. [IIED Issue Paper], London: IIED.

[R95] Raymond K (2012). Innovation to increase profitabiilty of steep terrain harvesting in New Zealand. New Zealand Journal of Forestry, 57, 19–23.

[R96] Raymond K (2014, November). New harvest technology for a safer future: Challenges on harvesting steepland. New Zealand Tree Grower, 3–6.

[R97] Review Panel. (2014). Independent forestry safety review: An agenda for change in the forestry sector. 12 p. Wellington, New Zealand: New Zealand Forest Owners Association.

[R98] Rhodes D (2001). Rehabilitation of deforested steep slopes on the East Coast of New Zealand’s North Island. Unasylva, 207, Article 4.

[R99] Richards K (2017). National Environmental Standard for Plantation Forests. Accessed 11 March 2021. https://nz.pfolsen.com/market-info-news/wood-matters/2017/october/national-environmental-standard-for-plantation-forests/

[R100] Richardson B, Vanner A, Ray J, Davenhill N, Coker G (1996). Mechanisms of *Pinus radiata* growth suppression by some common weed species. New Zealand Journal of Forestry Science, 26, 421–432.

[R101] Ross DJ, Scott NA, Lambie SM, Trotter CM, Rodda NJ, Townsend JA (2009). Nitrogen and carbon cycling in a New Zealand pumice soil under a manuka (*Leptospermum scoparium*) and kanuka (*Kunzea ericoides*) shrubland. Soil Research, 47, 725–736. 10.1071/SR08261

[R102] Ruff M, Czurda K (2008). Landslide susceptibility analysis with a heuristic approach in the Eastern Alps (Vorarlberg, Austria). Geomorphology, 94, 314–324. 10.1016/j.geomorph.2006.10.032

[R103] Sciarra M, Coco L, Urbano T (2017). Assessment and validation of GIS-based landslide susceptibility maps: a case study from Feltrino stream basin (Central Italy). Bulletin of Engineering Geology and the Environment, 76, 437–456. 10.1007/s10064-016-0954-7

[R104] Scott NA, White JD, Townsend JA, Whitehead D, Leathwick JR, Hall GMJ, Marden M, Rogers GND, Watson AJ, Whaley PT (2000). Carbon and nitrogen distribution and accumulation in a New Zealand scrubland ecosystem. Canadian Journal of Forest Research, 30, 1246–1255. 10.1139/x00-048

[R105] Soeters R, Van Westen CJ (1996). Slope stability: recognition, analysis and zonation. In: Turner AK, Shuster RL (Eds.), Landslides: investigation and mitigation, [Transportation Research Board Special Report 247], (pp. 129–177). Washington, USA, Transportation Research Board.

[R106] Spiekermann R, Marden M (2018). SLMACC 405415: Best options for land use following radiata harvest in the Gisborne District under climate change: Spatial analysis of erosion susceptibility in plantation forests, East Coast Region. [Landcare Research Contract Report LC3202], Wellington, New Zealand: Ministry for Primary Industries.

[R107] Stephens JMC, Molan PC, Clarkson BD (2005). A review of *Leptospermum scoparium* (*Myrtaceae*) in New Zealand. New Zealand Journal of Botany, 43, 431–449. 10.1080/0028825X.2005.9512966

[R108] Takatsuka Y, Cullen R, Wilson M, Wratten S (2009). Using stated preference techniques to value four key ecosystem services on New Zealand arable land. International Journal of Agricultural Sustainability, 7(4): 279–291. 10.3763/ijas.2009.0334

[R109] Tait A (2008). Future projections of growing degree days and frost in New Zealand and some implications for grape growing. Weather Climate, 28, 17–36. 10.2307/26169696

[R110] Tait A, Henderson R, Turner R, Zheng X (2006). Thin-plate smoothing spline interpolation of daily rainfall for New Zealand using a climatological rainfall surface. International Journal of Climatology, 26, 2097–2115. 10.1002/joc.1350

[R111] Tait A, Liley B (2009). Interpolation of daily solar radiation for New Zealand using a satellite-derived cloud cover surface. Weather and Climate, 29, 70–88. 10.2307/26169706

[R112] Te Tai Ōhanga. (2018). Discount rates. https://treasury.govt.nz/information-and-services/state-sector-leadership/guidance/financial-reporting-policies-and-guidance/discount-rates (Last accessed 25 June 2020).

[R113] Te Uru Rākau. (2020). Historic indicative New Zealand radiata pine log prices. Indicative prices of radiata pine logs by quarter since 1992. https://www.teururakau.govt.nz/news-and-resources/open-data-and-forecasting/forestry/wood-product-markets/historic-indicative-new-zealand-radiata-pine-log-prices/ (Last accessed 24 June 2020).

[R114] Tian Y, Wu H, Zhang G, Wang L, Zheng D, Li S (2020). Perceptions of ecosystem services, dissercies, and willingness-to-pay for urban green space conservation. Journal of Environmental Management, 260, 110140. 10.1016/j.jenvman.2020.110140 32090834

[R115] van Westen CJ, Castellanos E, Kuriakose SL (2008). Spatial data for landslide susceptibility, hazard, and vulnerability assessment: an overview. Engineering Geology, 102, 112–131. 10.1016/j.enggeo.2008.03.010

[R116] Varnes JD (1984). IAEG comission on landslides and other mass movements, landslide hazard zonation: a review of principles and practice. pp 63. Paris, France: UNESCO Press.

[R117] Veld GJ, de Graaf F (1990). Erosion damage as a result of Cyclone Bola: an assessment on Arai Matawai and Emerald Hills properties East Coast Region, North Island, New Zealand. Gisborne, 41 p. New Zealand: Forest Research Institute and the Ministry of Forestry.

[R118] Walsh PJ, Soliman T, Greenhalgh S, Mason NWH, Palmer D (2017). Valuing the benefits of permenant forests. [Ministry for Primary Industries Technical Paper No: 2017/68], Wellington, New Zealand: Ministry for Primary Industries.

[R119] Walsh P, Soliman T, Robertson T (2019). A cost-benefit analysis of transitions from exotic to native forestry in Gisborne. [Landcare Research Ccontract Report LC3553] Lincoln, New Zealand: Landcare Research.

[R120] Watt MS, Kimberley MO, Coker G, Richardson B, Estcourt G (2007). Modelling the influence of weed competition on growth of young *Pinus radiata*. Development and parametization of a hybrid model across an environmental gradient. Canadian Journal of Forest Research, 37, 607–616. 10.1139/X06-254

[R121] Watt MS, Kirschbaum MUF, Meason D, Jovner A, Pearce HG, Moore JR, Nicholas I, Bulman L, Rolando C, Palmer DJ, Harrison D, Hock BK, Tait A, Ausseil A-GE (2012). Future Forest Systems. 180 p. [Scion Client Report]. Rotorua, New Zealand: Scion.

[R122] Wetere W (2015). What’s *our potential? Maniapoto and manuka honey*. Presentation at Ngā Aho Rangahau o Maniapoto - Threads of Research Symposium, Te Kuiti, July 2015.

[R123] Whitehead D, Walcroft AS (2005). Forest and shrubland canopy carbon uptake in relation to foliage nitrogen concentration and leaf area index: A modelling analysis. Annals of Forest Science, 62, 525–535. 10.1051/forest:2005045

[R124] Whitehead D, Walcroft AS, Scott NA, Townsend JA, Trotter CM, Rogers G, Rogers GND (2004). Characteristics of photosynthesis and stomatal conductance in the shrubland species mānuka (*Leptospermum scoparium*) and kānuka (*Kunzea ericoides*) for the estimation of annual canopy carbon uptake. Tree Physiology, 24, 795–804. 10.1093/treephys/24.7.795 15123451

[R125] Williams PA (1983). Secondary vegetation succession on the Port Hills, Banks Peninsula, Canterbury, New Zealand. New Zealand Journal of Ecology, 21, 237–247. 10.1080/0028825X.1983.10428556

[R126] Wilson HD (1994). Regeneration of native forest on Hinewai Reserve, Banks Peninsula. New Zealand Journal of Ecology, 32, 373–383. 10.1080/0028825X.1994.10410480

[R127] Yao RT, Scarpa R, Turner JA, Barnard TD, Rose JM, Palma JHN, Harrison DR (2014). Valuing biodiversity enhancement in New Zealand’s planted forests: Socioeconomic and spatial determinants of willingness-to-pay. Ecological Economics, 98, 90–101. 10.1016/j.ecolecon.2013.12.009

[R128] Yalcin A, Bulut F (2007). Landslide susceptibility mapping using GIS and digital photogrammetric techniques: a case study from Ardesen (NETurkey). Natural Hazards, 41, 201–226. 10.1007/s11069-006-9030-0

